# Supplementary material for: Consumption of High-Energy Food and Sugar Shows a Strong Positive Association with Low Mood in Control Subjects and Depressed Patients
Source: Nutrients. 2025 Aug 9;17(16):2594. doi: 10.3390/nu17162594 (PMC12389405; doi:10.3390/nu17162594)
Supplement: Supplementary file 1 [file nutrients-17-02594-s001.zip › nutrients-3767929-Supplementary Table S1.pdf]

Consumption of high-energy food and sugar shows strong positive association with low mood in control subjects and depressed patients

Tabita Dobai [1,2,†], Daniel Baksa [1,2,3,4,†], Xenia Gonda [1,2,4,5,6], Gabriella Juhasz [1,2,4,], Nora Eszlari [1,2,4, ‡] and Gyorgy Bagdy [1,2,4,*,‡]

[1] Department of Pharmacodynamics, Faculty of Pharmaceutical Sciences, Semmelweis University, Nagyvárad tér 4, H-1089 Budapest, Hungary;
[2] Center of Pharmacology and Drug Research & Development, Semmelweis University, Üllői Street 26, H-1085 Budapest, Hungary;
[3] Department of Personality and Clinical Psychology, Institute of Psychology, Faculty of Humanities and Social Sciences, Pazmany Peter Catholic University, Mikszáth Kálmán tér 1, H-1088 Budapest, Hungary;
[4] NAP3.0-SE Neuropsychopharmacology Research Group, Hungarian Brain Research Program, Semmelweis University, Üllői Street 26, H-1085 Budapest, Hungary;
[5] Department of Psychiatry and Psychotherapy, Semmelweis University, Gyulai Pál utca 2, H-1085 Budapest, Hungary;
[6] Department of Clinical Psychology, Semmelweis University, Üllői út 25, H-1091 Budapest, Hungary;
* Correspondence: bagdy.gyorgy@semmelweis.hu ; Tel.: +36-1459-1500 (ext. 56331)
† These authors contributed equally to this work as first authors
‡ These authors contributed equally to this work as last authors

Supplementary Table S1. Description of our study variables, depicting their data acquisition points, and ordered by data acquisition date.

Data Field:	Name:	Data acquisition	Instance 0	Instance 1	Instance 2	Instance 3	Instance 4	Comment
189-0.0	Townsend deprivation index	Calculated immediately prior to participant joining UK Biobank; and each participant's score corresponds to the output area in which their postcode is located.						
130894-0.0 and 130896-0.0	ICD-10 F32orF33	Electronic Health Record	Date F32 or Date F33 first reported					
21003-0.0	age	Initial assessment visit	2006 to 2010					
21001-0.0	BMI							
2050-0.0	depressed_mood (from the four-item depression questionnaire)	Initial assessment visit	2006 to 2010					
2060-0.0	loss_of_interest (from the four-item depression questionnaire)							
2070-0.0	restlessness (from the four-item depression questionnaire)							
2080-0.0	tiredness (from the four-item depression questionnaire)							
26002	Energy	Instance 0: Conducted in assessment centre Instance 1: On-	Apr 2009 to Sept 2010	Feb 2011 to Ap 2011	Jun 2011 to Sep 2011	Oct 2011 to Dec 2011	Apr 2012 to Jun 2012	In our study, averaged for each individual
26004	Energy density							
26003	Energy from beverages							

26001	Total weight of beverages only	line cycle 1 Instance 2: On-line cycle 2 Instance 3: On-line cycle 3 Instance 4: On-line cycle 4							between all answered data acquisition points (out of the five)
26013	Carbohydrate								
26012	Free sugar								
26044	Fructose								
26045	Glucose								
26050	Intrinsic and milk sugars								
26048	Lactose								
26049	Maltose								
26055	Non-milk extrinsic sugars								
26056	Other Sugars								
26059	Sucrose								
31-0.0	Sex	Acquired from central registry at recruitment, but in some cases updated by the participant	Jan 2012 to Mar 2022						
20514-0.0	PHQ9_lack_of_interest	Online follow-up	Oct 2016 to Jul 2017						
20510-0.0	PHQ9_depressed_mood								
20517-0.0	PHQ9_sleep								
20519-0.0	PHQ9_tiredness								
20511-0.0	PHQ9_appetite								
20507-0.0	PHQ9_inadequacy								
20508-0.0	PHQ9_concentrating								
20518-0.0	PHQ9_speed								
20513-0.0	PHQ9_suicide								
20446	CIDI - Ever had prolonged feelings of								

	sadness or depression							
20441	CIDI - Ever had prolonged loss of interest in normal activities							
20536	CIDI - Weight change during worst episode of depression							
20532	CIDI - Did your sleep change?							
20449	CIDI - Feelings of tiredness during worst episode of depression							
20450	CIDI - Feelings of worthlessness during worst period of depression							
20435	CIDI - Difficulty concentrating during worst depression							
20437	CIDI - Thoughts of death during worst depression							

Based on UK Biobank Data Showcase: https://biobank.ndph.ox.ac.uk/showcase/. ICD: International Classification of Diseases, BMI: body mass index, PHQ9: Patient Health Questionnaire 9, CIDI: Composite International Diagnostic Interview, grey shading: instance number is not relevant for that variable.
